# The Impact of High-Temperature and High-Pressure Steaming Treatment on Bioactive Compounds and Antioxidant Activity in American Ginseng

**DOI:** 10.3390/molecules31203706

**Published:** 2026-10-16

**Authors:** Wenhui Yu, Ruoqi Han, Taihong Liu, Xuemei Sun, Han Wang, Juan Fu, Shucheng Duan, Seok Hyun Eom, Chengwu Jin

**Affiliations:** 1 School of Food Engineering, Yantai Engineering Research Center of Green Food Processing and Quality Control, Ludong University, Yantai 264025, China; 13127121684@163.com (W.Y.); kwsxhrq@163.com (R.H.); 18561029246@163.com (T.L.); 2875@ldu.edu.cn (X.S.); 2 College of Biological and Pharmaceutical Engineering, Shandong University of Aeronautics, Binzhou 256600, China; whsunny0202@163.com (H.W.); sparrowfj@126.com (J.F.); dsc97@sdua.edu.cn (S.D.); 3 Binzhou Key Laboratory of Edible Fungi Breeding and High Value Utilization (Preparation), College of Biological and Pharmaceutical Engineering, Shandong University of Aeronautics, Binzhou 256600, China; 4 Graduate School of Green-Bio Science, College of Life Sciences, Kyung Hee University, Yongin 17104, Republic of Korea; se43@khu.ac.kr

**Keywords:** thermal processing, ginsenoside, conversion, bioactivity

## Abstract

Thermal processing facilitates the transformation of major ginsenosides into rare ginsenosides, thereby enhancing the bioactive molecular profile of American ginseng. This study examined the impact of 1–4 cycles of high-temperature and high-pressure steaming (HTHPS) on ginsenoside composition, total phenolic content (TPC), total flavonoid content (TFC), and antioxidant activity in American ginseng. HTHPS significantly decreased major ginsenoside levels while increasing the content of rare ginsenosides. Following three cycles (HTHPS-3), the concentrations of Rg3, Rk1, Rg5, Rh1, F4, and Rg6 reached 8.68, 6.02, 20.07, 1.92, 3.72, and 2.57 mg/g, respectively, in contrast to their absence in the untreated control. TPC exhibited a 6.74-fold increase after HTHPS-2, whereas TFC showed a 4.27-fold increase following HTHPS-3. Pearson correlation analysis revealed strong positive correlations between rare ginsenosides, TPC, TFC, and antioxidant activity. In summary, HTHPS treatment significantly altered the bioactive profile of American ginseng. HTHPS-2 was more suitable for maximizing TPC and DPPH/ABTS^+^ radical-scavenging activities, whereas HTHPS-3 favored rare ginsenoside accumulation, particularly Rg3, Rk1, and Rg5, and exhibited a stronger ·OH radical-scavenging capacity. These findings indicate that the preferred number of HTHPS cycles depends on the target bioactive components and functional properties.

## 1. Introduction

American ginseng (*Panax quinquefolius* L.) represents a significant medicinal and nutritional resource, characterized by its diverse array of bioactive compounds, including ginsenosides, polyphenols, and flavonoids. These compounds exhibit a range of health-promoting activities, such as antioxidant and immunomodulatory effects [1,2,3].

Ginsenosides, the primary bioactive constituents of American ginseng, are categorized into protopanaxadiol (PPD) and protopanaxatriol (PPT) types based on their aglycone structures. In unprocessed American ginseng, major ginsenosides like Rb1, Re, and Rd are predominant, while rare ginsenosides, such as Rg3 and Rh2, are typically present in lower concentrations. The unique biological activities of these rare ginsenosides have garnered significant interest [4]. In particular, rare ginsenosides such as Rg3, Rg5, Rk1, Rh1, F4, and Rg6 possess antitumor, anti-inflammatory, and immunomodulatory activities and have high bioavailability [5]; due to their low natural abundance and high cost, they have significant commercial value. Consequently, converting major ginsenosides into rare ginsenosides represents an important strategy for enhancing the value of processed American ginseng by increasing the abundance of bioactive molecules.

Thermal processing, especially moist-heat treatments like steaming, can enhance major ginsenoside conversion through hydrolysis and deglycosylation, thereby facilitating the production of rare ginsenosides in *Panax* species [6,7,8,9]. However, conventional steaming often requires 4–9 cycles over more than 60 h, resulting in prolonged processing and potentially limiting processing efficiency [10]. Although alternative techniques have been explored, each has its own limitations. Far-infrared heating offers high conversion efficiency, it carries a significant risk of thermal degradation at high temperatures [11]. Microwave-assisted processing is limited by penetration depth, making it difficult to achieve uniform processing on a large scale [12]. Subcritical water extraction offers high extraction efficiency but requires high-pressure conditions of 5–20 MPa and exhibits poor selectivity at high temperatures. Furthermore, excessive heating or prolonged processing times may lead to the degradation of heat-sensitive bioactive compounds [13]. These constraints may impede the advanced processing and high-value utilization of American ginseng.

HTHPS integrates heat, pressure, and moisture; using saturated steam as a medium under mild conditions, wet-heat synergistic hydrolysis is expected to promote major ginsenoside conversion and deglycosylation, thereby facilitating rare ginsenoside formation [14,15]. Additionally, HTHPS may disrupt plant cell structures, minimizing the thermal degradation of heat-sensitive bioactive compounds and thereby potentially promoting the release of phenolic and flavonoid compounds. However, repeated application of this treatment regimen may further degrade or modify these bioactive compounds [16,17].

**Figure 1:**
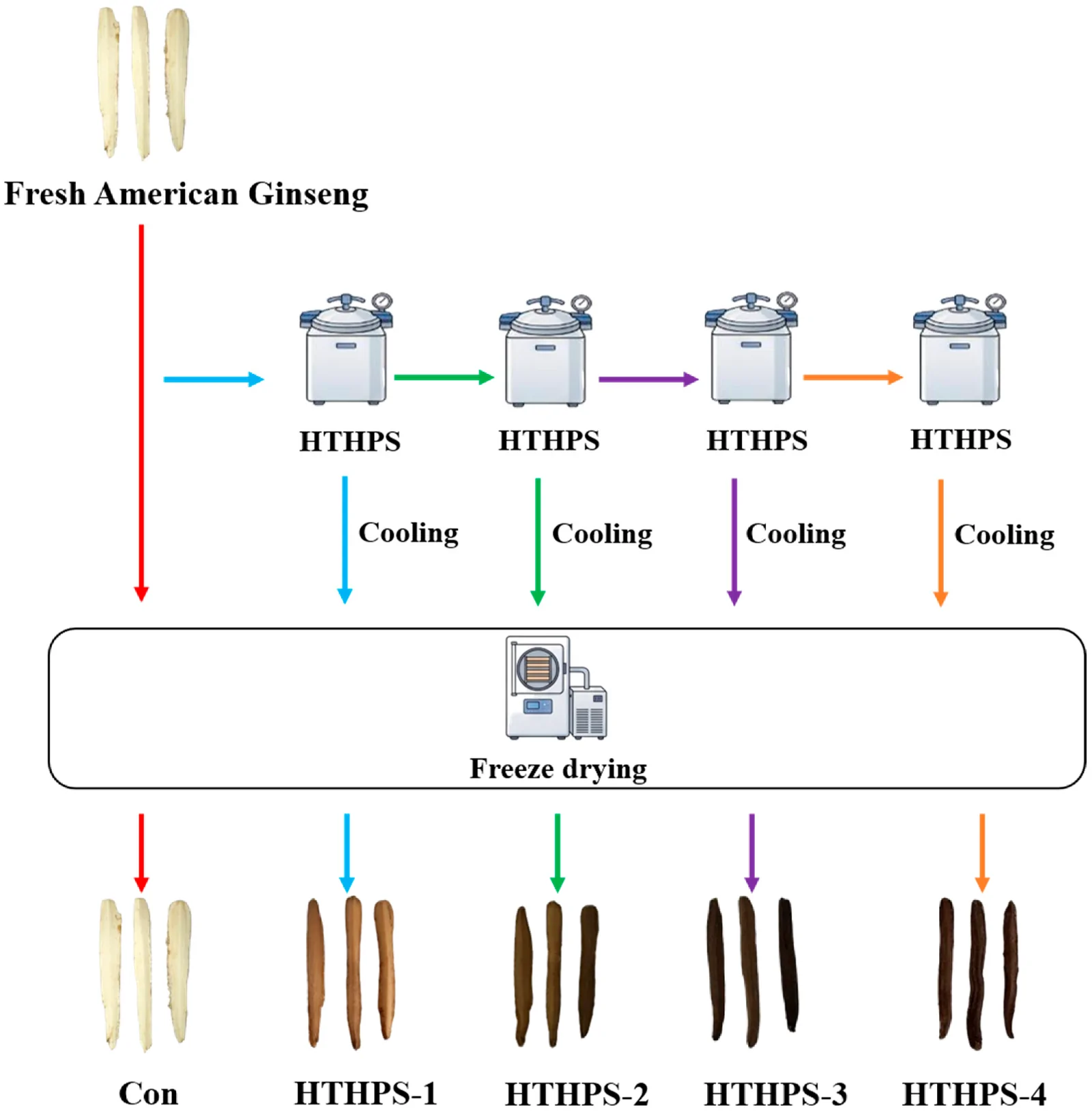
Schematic diagram of the experimental procedure and morphological characteristics of the sample after HTHPS treatment.

Considering the information mentioned above, American ginseng underwent 1–4 cycles of intermittent HTHPS treatment. The experimental procedure is illustrated in Figure 1. The investigation focused on alterations in individual PPD- and PPT-type major and minor ginsenosides, and TPC and TFC were also investigated. Antioxidant activity was assessed by measuring DPPH, ABTS^+^, and ·OH radical-scavenging activities to evaluate the impact of HTHPS. Pearson correlation analysis was conducted to explore the relationships between bioactive components and antioxidant indices. The objective was to identify an optimal HTHPS cycle to enhance the bioactive profile of American ginseng.

## 2. Results and Discussion

### 2.1. Morphology and Color Parameters

**Figure 2:**
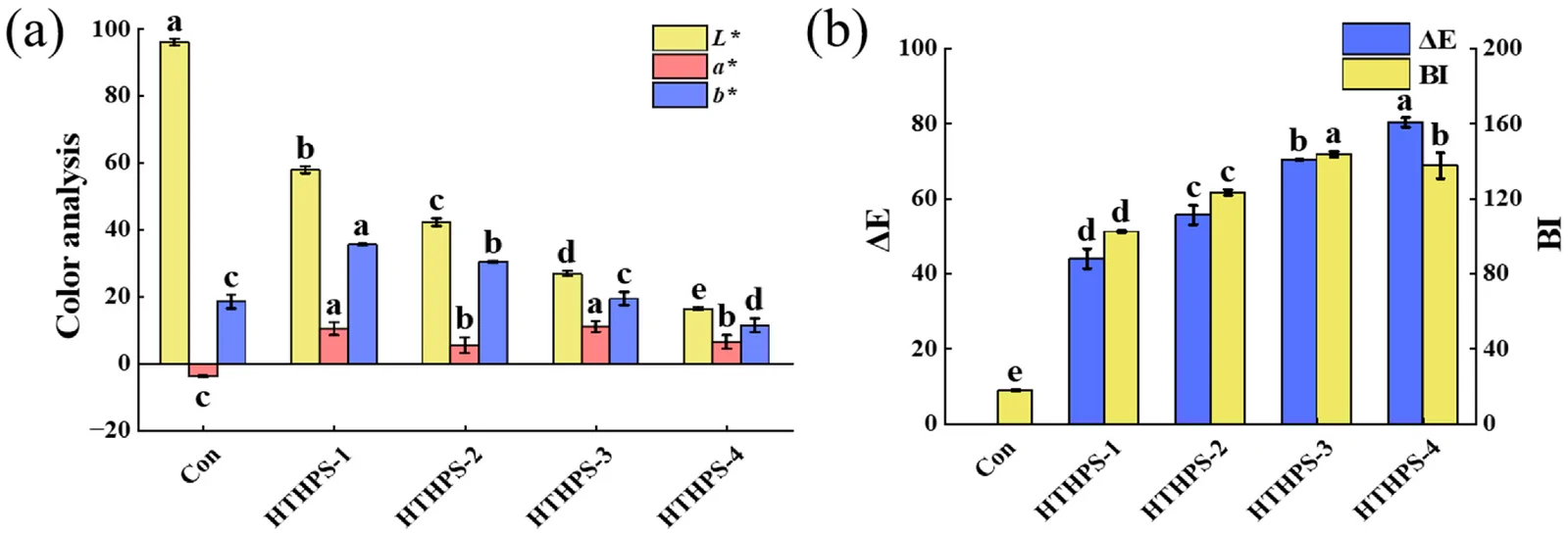
Effect of HTHPS on the color parameters in American ginseng: (**a**) color analysis and (**b**) ΔE and BI. Different lowercase letters in the same color column indicate significant differences among treatments (*p* < 0.05) on Tukey’s HSD.

Figure 1 and Figure 2 illustrate the morphological characteristics and color parameters of American ginseng after different cycles of HTHPS treatment. The control sample exhibited a pale yellow-white appearance, whereas HTHPS application resulted in a progressive darkening of color, transitioning from light yellow to dark brown (Figure 1). These visual transformations were corroborated by color parameter analysis. Specifically, the L* value decreased with successive HTHPS cycles, indicating a gradual reduction in brightness. Concurrently, alterations in the a* and b* values signified a shift in color from the initial light yellow-white to a darker brown hue (Figure 2a). The overall color difference (ΔE) exhibited a significant increase post-HTHPS, with further increments observed as the number of treatment cycles increased (Figure 2b), thereby indicating substantial color modification. A similar upward trend was noted for the BI, suggesting that repeated HTHPS facilitated the formation of brown-colored compounds. The browning observed during treatment may be associated with several possible processes, including Maillard and caramelization reactions, as well as the degradation or transformation of endogenous pigments and phenolic compounds [18,19,20].

### 2.2. Ginsenoside Contents

**Figure 3:**
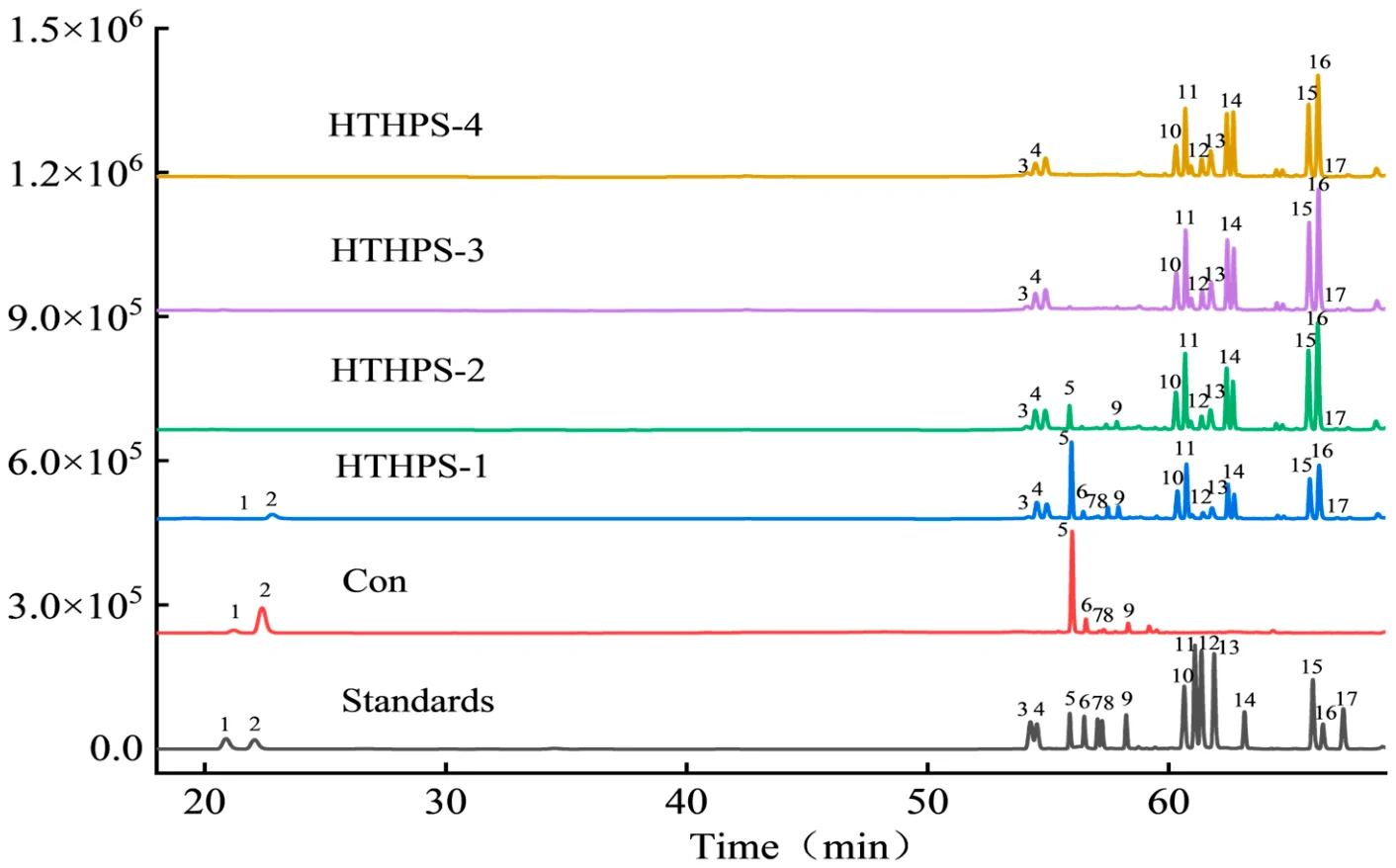
HPLC chromatogram of ginsenosides (1-Rg1, 2-Re, 3-Rh1, 4-Rg2, 5-Rb1, 6-Rc, 7-Rb2, 8-Rb3, 9-Rd, 10-Rg6, 11-F4, 12-Rk3, 13-Rh4, 14-Rg3, 15-Rk1, 16-Rg5, and 17-Rh2).

**Figure 4:**
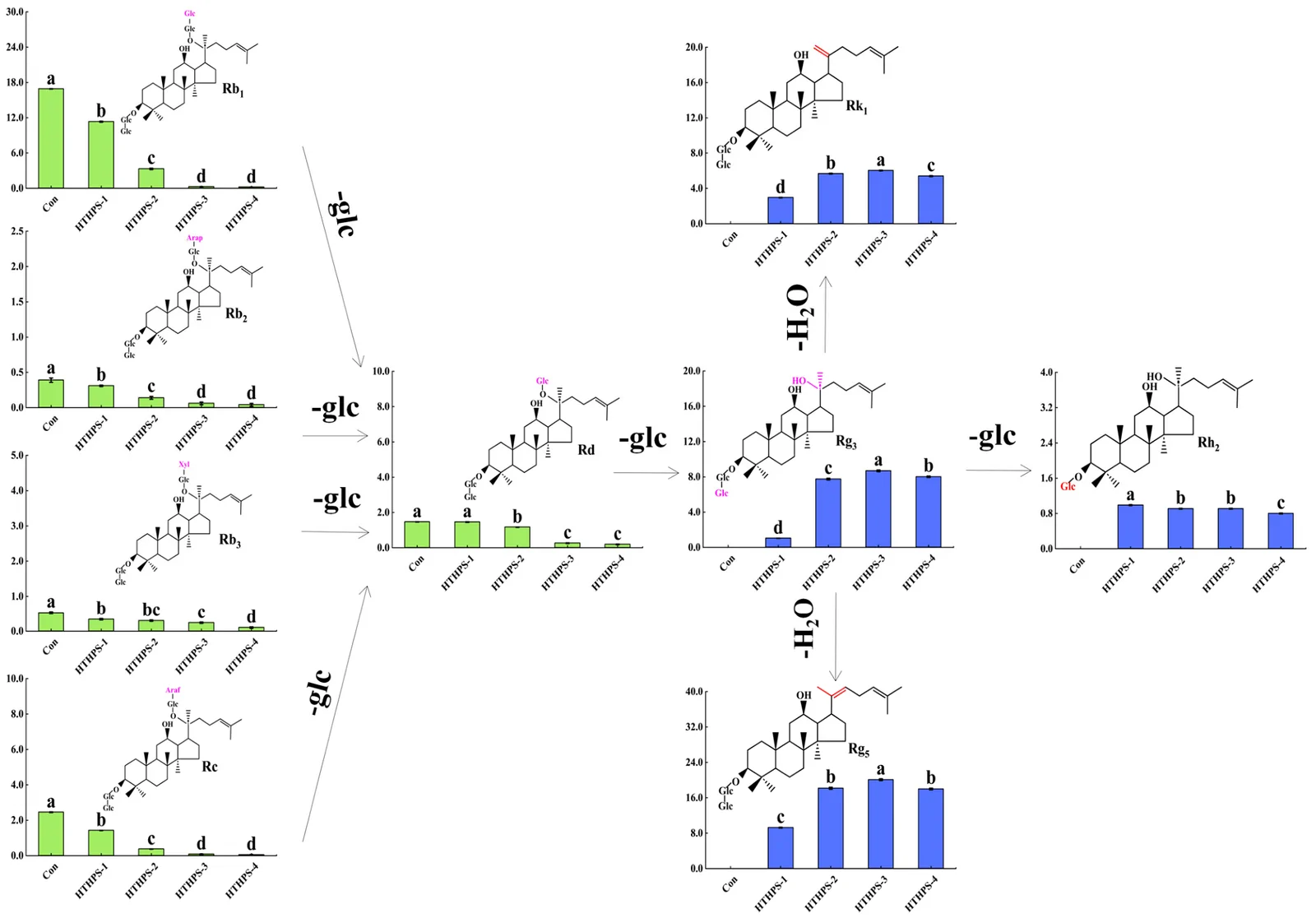
Effects of HTHPS on the contents of PPD-type ginsenosides in American ginseng and proposed transformation pathways. The different lowercase letters (a–d) above the column indicate significant differences (*p* < 0.05) among treatments on Tukey’s HSD. The green and blue column indicate major and minor ginsenosides, respectively.

**Figure 5:**
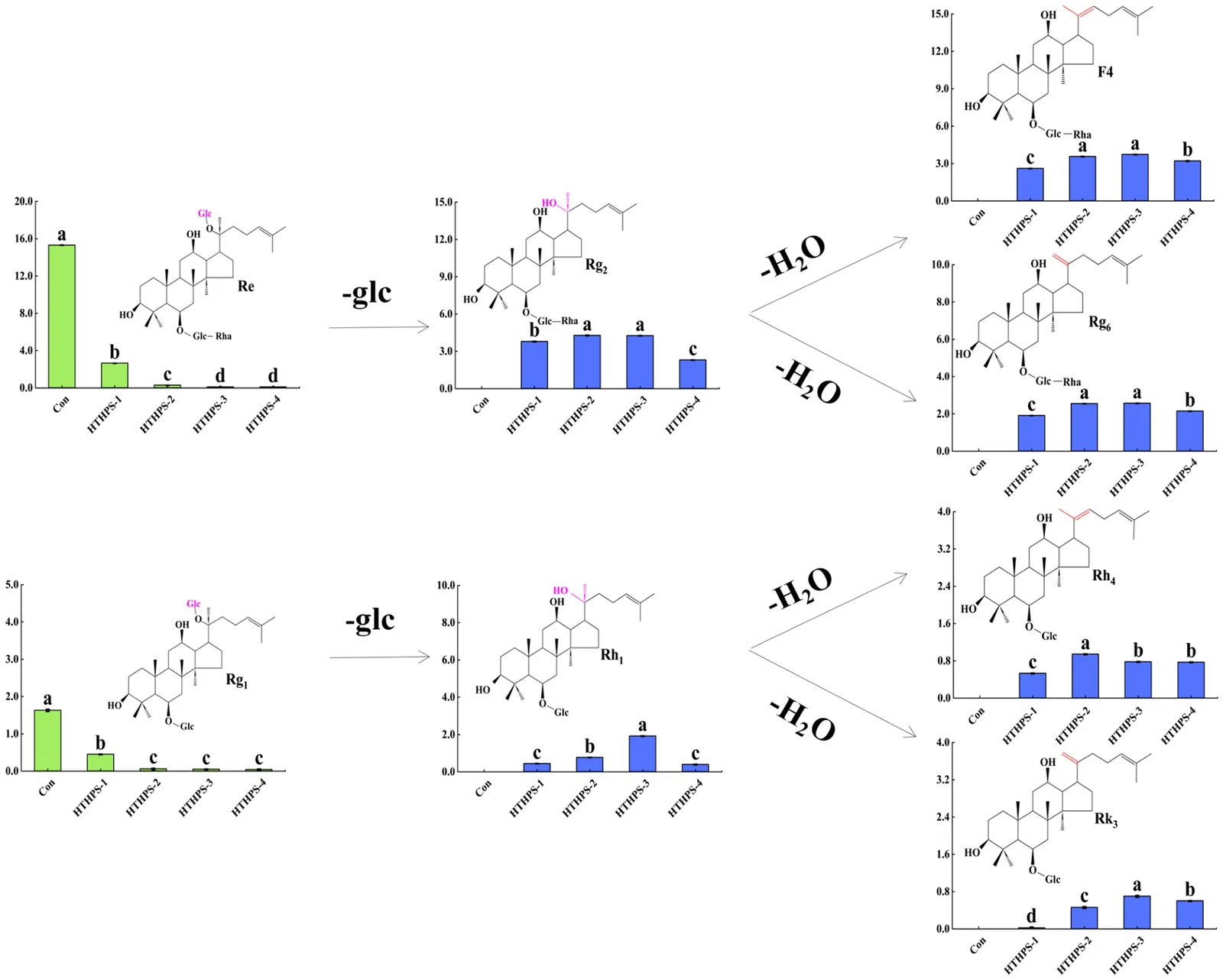
Effects of HTHPS on the contents of PPT-type ginsenosides in American ginseng and proposed transformation pathways. The different lowercase letters (a–d) above the column indicate significant differences (*p* < 0.05) among treatments on Tukey’s HSD. The green and blue column indicate major and minor ginsenosides, respectively.

HTHPS markedly changed the ginsenoside profile of American ginseng (Figure 3). The contents of major ginsenosides decreased with increasing HTHPS cycles (Figure 4 and Figure 5). In contrast, most rare ginsenosides increased initially and then declined slightly after HTHPS-3.

#### 2.2.1. PPD-Type Ginsenosides

Figure 4 illustrates the alterations in PPD-type ginsenosides during HTHPS. The principal ginsenosides, namely Rb1, Rb2, Rb3, and Rc, exhibited a progressive decline in concentration with an increasing number of treatment cycles. In the control sample, Rb1 was the most prevalent PPD-type ginsenoside, measured at 16.91 mg/g, followed by Rc at 2.45 mg/g. After three HTHPS cycles, their concentrations diminished to 0.26 and 0.08 mg/g, respectively, and both remained at reduced levels after HTHPS-4.

Rd displayed a distinct pattern. Its concentration remained relatively stable between the control and HTHPS-1. It then decreased to 1.17 mg/g after HTHPS-2 and to about 0.20 mg/g after HTHPS-3 and HTHPS-4. Overall, repeated HTHPS markedly reduced the major PPD-type ginsenosides.

The ginsenosides Rg3, Rk1, Rg5, and Rh2 were not detected in the control but appeared after HTHPS. Specifically, Rg3, Rk1, and Rg5 attained their peak concentrations after HTHPS-3, measuring 8.68, 6.02, and 20.07 mg/g, respectively. Rh2 reached 0.99 mg/g after HTHPS-1. Most rare ginsenosides decreased slightly after HTHPS-3; they remained elevated compared to the control. Rg5 was the most abundant rare PPD-type ginsenoside throughout the treatment process.

Previous studies have proposed that Rd may act as an intermediate in the conversion of major PPD-type ginsenosides, including Rb1, Rc, Rb2, and Rb3, into rare ginsenosides such as Rg3, Rk1, and Rg5 [21]. In the present study, the changes in ginsenoside contents during HTHPS treatment were generally consistent with the transformation patterns reported previously. As the number of steaming cycles increased, the contents of the major PPD-type ginsenosides (Rb1, Rc, Rb2, and Rb3) and Rd decreased, whereas those of Rg3, Rk1, Rg5, and Rh2 initially increased and subsequently decreased. Among the rare ginsenosides, Rg5 exhibited the highest content (20.07 mg/g), followed by Rg3 (8.68 mg/g), with both reaching their maximum levels in the HTHPS-3 group. These results demonstrate substantial changes in the PPD-type ginsenoside profile during HTHPS treatment, with several rare ginsenosides reaching their highest measured contents after three steaming cycles. Overall, the observed concentration patterns are consistent with the deglycosylation and dehydration reactions proposed in previous studies [22]. Under the conditions tested, HTHPS-3 was the most favorable treatment for maximizing the measured contents of several rare PPD-type ginsenosides.

#### 2.2.2. PPT-Type Ginsenosides

The application of HTHPS also influenced the composition of PPT-type ginsenosides (Figure 5). In the Con, Re and Rg1 were the predominant PPT-type major ginsenosides, with contents of 15.30 and 1.63 mg/g, respectively. However, their levels significantly declined with successive HTHPS cycles. The rare ginsenosides Rh1, Rg2, Rg6, F4, Rk3, and Rh4 were not detected in the Con but were generated after HTHPS treatment. Rg2 increased from 3.79 mg/g in HTHPS-1 to 4.27 mg/g in HTHPS-2 and remained relatively stable in HTHPS-3 (4.25 mg/g). F4 and Rg6 reached maximum levels of 3.72 and 2.57 mg/g, respectively, in HTHPS-3. Rh1 continued to increase and reached its highest level of 1.92 mg/g. Rk3 also reached its maximum level (0.70 mg/g) in HTHPS-3. After HTHPS-4, most rare ginsenosides decreased.

Following HTHPS treatment, the PPT-type rare ginsenosides Rh1, Rg2, Rg6, F4, Rk3, and Rh4 were detected. Tang et al. [23] found that treating ground ginseng at 135 °C and 103 kPa for 30 min promoted Rg2 and Rh1 formation, whereas F4, Rg6, Rh4, and Rk3 were not detected. These findings suggest that the extent of ginsenoside transformation may depend on the processing conditions. Zhang et al. [24] reported detecting Rg6 and F4 following autoclave treatment of American ginseng roots, with contents of 0.68 and 0.55 mg/g, respectively. In the present study, their contents reached 2.57 mg/g and 3.72 mg/g, respectively, in the HTHPS-3 group.

The contents of the major PPT-type ginsenosides Rg1 and Re decreased markedly after steaming, with reductions exceeding 80% in the HTHPS-1 group and 99% in the HTHPS-3 group relative to the control. This trend was consistent with the observations reported by Liu et al. [25] and Yao et al. [26]. In addition, the contents of the detected rare PPT-type ginsenosides initially increased and subsequently decreased with increasing steaming cycles. Collectively, these results demonstrate pronounced changes in the PPT-type ginsenoside profile during HTHPS treatment, with HTHPS-3 yielding the highest measured contents of the rare ginsenosides examined.

The findings indicate that HTHPS facilitated the sequential transformation of PPT-type ginsenosides. Two principal transformation pathways are proposed: Re→Rg2→F4/Rg6 and Rg1→Rh1→Rh4/Rk3 (Figure 4). The initial phase involves deglycosylation (−Glc), which is succeeded by dehydration of the intermediate ginsenosides. The emergence of Rg2, Rh1, Rg6, F4, Rk3, and Rh4 following treatment aligns with the existing literature, which suggests that thermal treatment enhances major PPT-type ginsenoside conversion into rare ginsenosides [27].

Overall, the present results indicate that repeated HTHPS treatment induces a sequential, cycle-dependent transformation of ginsenosides. Initially, major ginsenosides are converted through deglycosylation into intermediate compounds, which are subsequently subjected to further deglycosylation and dehydration, resulting in downstream rare ginsenoside formation. The distinct accumulation patterns of individual ginsenosides imply that these reactions occur in a sequential manner rather than simultaneously. Following HTHPS-4, the observed decline in most rare ginsenosides indicates a transition from product formation to further transformation and/or degradation. Our previous research has established that increasing temperature is a major factor driving ginsenoside deglycosylation, dehydration, and subsequent structural degradation in ginseng leaves [28]. In contrast, the current study was conducted under a constant thermal condition, demonstrating that the number of heating cycles controls ginsenoside conversion progression. Specifically, HTHPS-3 appears to strike an optimal balance between precursor conversion and rare ginsenoside accumulation.

#### 2.2.3. Total Ginsenoside Content

**Figure 6:**
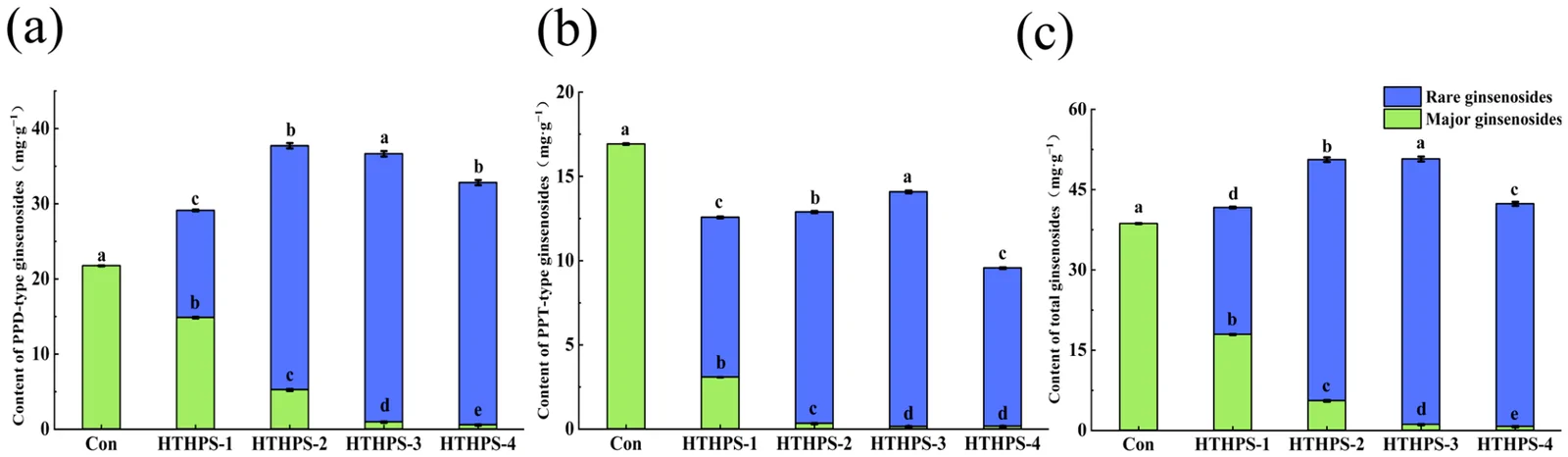
Effect of HTHPS on the content of (**a**) PPD-type, (**b**) PPT-type, and (**c**) total ginsenosides in American ginseng. The different lowercase letters (a–e) above the column indicate significant differences (*p* < 0.05) test among treatments on Tukey’s HSD.

As shown in Figure 6, total ginsenoside content and composition changed significantly with increasing HTHPS cycles. In the Con, PPD- and PPT-type ginsenosides were at 21.75 and 16.92 mg/g (Figure 6a,b), respectively, with a total content of 38.67 mg/g (Figure 6c). After HTHPS, major ginsenosides decreased markedly, whereas rare ginsenosides accumulated. Total ginsenoside content peaked at 49.62 mg/g in HTHPS-3 before declining to 41.64 mg/g in HTHPS-4 (Figure 6c). These changes suggest that repeated HTHPS promoted major ginsenoside transformation into rare ginsenosides, while prolonged treatment may have increased their subsequent degradation. The proposed hydrolysis, deglycosylation, and dehydration reactions are consistent with previous reports [29,30]. Overall, HTHPS-3 yielded the highest total ginsenoside content under the conditions tested.

### 2.3. TPC and TFC

**Figure 7:**
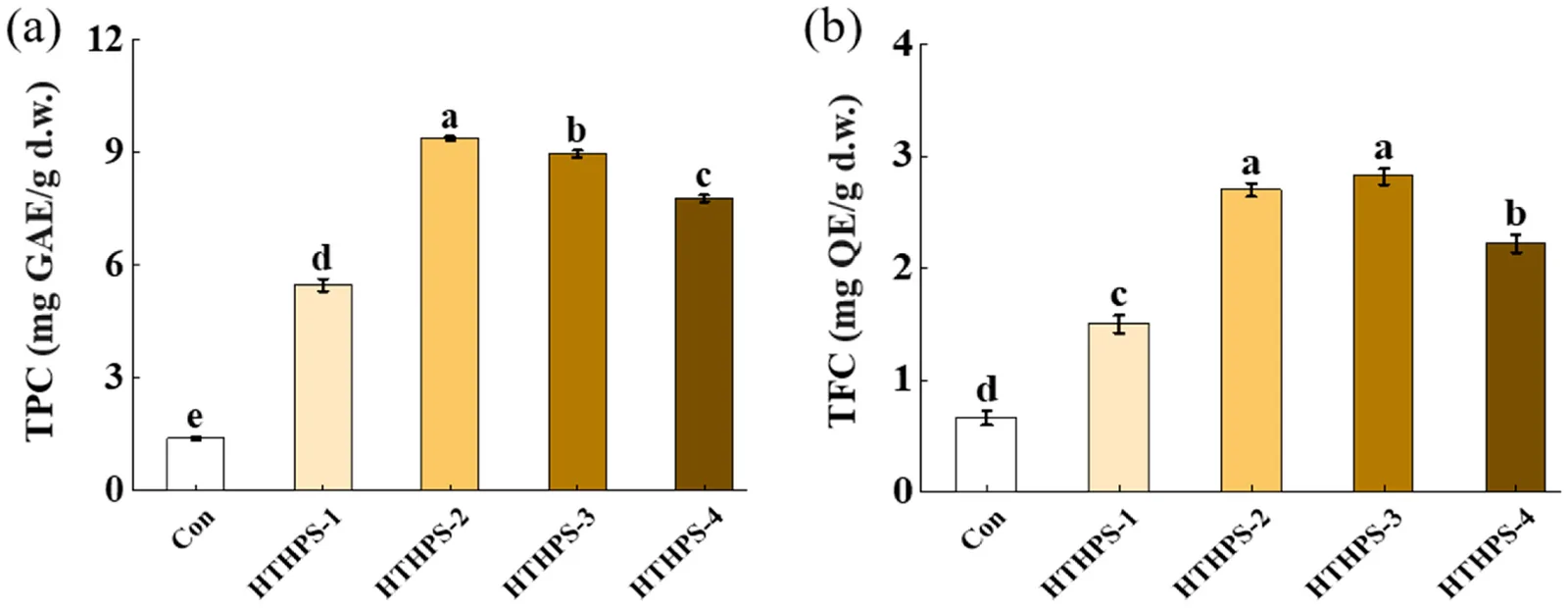
Effect of HTHPS on the TPC (**a**) and TFC (**b**) in American ginseng. The different lowercase letters (a–e) above the column indicate significant differences (*p* < 0.05) among treatments on Tukey’s HSD.

HTHPS significantly affected the TPC and TFC of American ginseng (Figure 7). TPC increased from 1.39 mg/g in the control to 5.47, 9.37, 8.95, and 7.77 mg/g after 1–4 HTHPS cycles, respectively (Figure 7a). TPC reached its highest level after HTHPS-2. TFC showed a similar trend. It increased from 0.66 mg/g in the control to 1.50, 2.70, and 2.82 mg/g after HTHPS-1, HTHPS-2, and HTHPS-3, respectively. It then decreased to 2.22 mg/g after HTHPS-4 (Figure 7b). Both TPC and TFC remained significantly higher than the control after HTHPS treatment.

The initial increases in TPC and TFC during HTHPS treatment may be associated with several processing-induced effects. Heat and pressure may alter cell wall structure and disrupt interactions between cell wall components and phytochemicals, thereby improving phenolic compound extractability [31]. Some phenolics occur in soluble conjugated or insoluble bound forms associated with cell wall structural components. Thermal processing may facilitate the release of these compounds through matrix disruption or chemical bond cleavage, potentially increasing their recovery during extraction. In addition, thermal reactions, including the Maillard reaction, may generate reducing substances such as melanoidins that contribute to antioxidant activity and potentially interfere with colorimetric assays [32,33,34,35]. Nonetheless, extended treatment duration may lead to the thermal degradation or oxidation of certain phenolic and flavonoid compounds [36,37]. TPC began to decline after HTHPS-2, whereas TFC remained relatively high until HTHPS-3. This finding may reflect differences in their thermal stability.

### 2.4. Antioxidant Activity

Table 1 shows the effect of HTHPS on the DPPH, ABTS^+^, and ·OH radical-scavenging activities (IC_50_) of American ginseng. All three antioxidant assays showed a consistent trend: an initial increase in activity followed by a slight decline as the treatment period increased, although the peak response occurred at different stages. DPPH radical-scavenging activity (IC_50_) decreased from 22.35 mg/mL in the Con to 6.22 mg/mL and 3.50 mg/mL in the HTHPS-1 and HTHPS-2 groups, respectively, and subsequently dropped to 3.89 mg/mL and 4.34 mg/mL in the HTHPS-3 and HTHPS-4 groups. Similarly, ABTS^+^ scavenging activity (IC_50_) also decreased significantly from 4.22 mg/mL in the Con to 2.06 mg/mL in the HTHPS-1 group, peaked at 1.47 mg/mL in the HTHPS-2 group, and then gradually increased. ·OH scavenging activity (IC_50_) decreased from 30.08 mg/mL in the Con to 11.52 mg/mL, 10.27 mg/mL, and 9.56 mg/mL in the HTHPS-1, HTHPS-2, and HTHPS-3 groups, respectively, and then increased slightly to 10.05 mg/mL in the HTHPS-4 group.

The enhanced antioxidant activity during early HTHPS was consistent with the increases in bioactive compound contents, like rare ginsenosides, polyphenols, and flavonoids (Figure 2, Figure 3, Figure 4, Figure 5 and Figure 6), implying that HTHPS promoted the release and/or transformation of antioxidant compounds [38,39,40,41,42]. The strongest DPPH and ABTS^+^ scavenging activities were observed in HTHPS-2, whereas ·OH scavenging activity was achieved at HTHPS-3. These variations indicate that the contribution of bioactive compounds to antioxidant activity may differ depending on the specific radical species involved.

### 2.5. Correlation Analysis Between Bioactive Components and Antioxidant Activity

**Figure 8:**
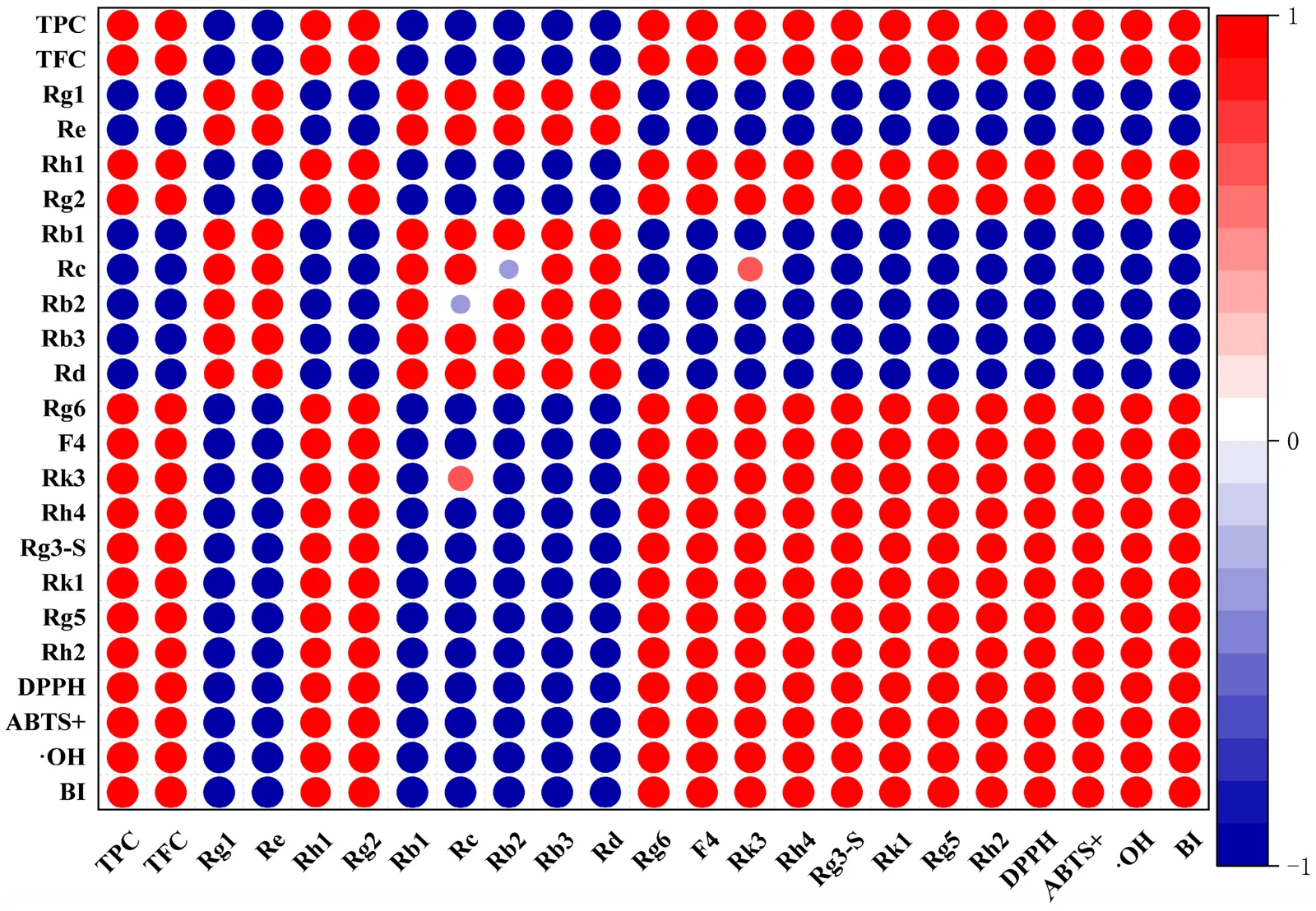
Pearson correlation analysis between bioactive compounds and antioxidant activities in American ginseng. The analysis was performed using the mean values of the five treatment groups (Con and HTHPS-1 to HTHPS-4; N = 5).

Pearson correlation analysis identified distinct associations between bioactive components and antioxidant activity (Figure 8). TPC and TFC showed strong positive correlations with DPPH, ABTS^+^, and ·OH radical-scavenging activities (*p* < 0.01). Several rare ginsenosides, particularly Rg6, F4, Rk1, and Rg5, also showed strong positive correlations with antioxidant activity (*p* < 0.01), whereas major ginsenosides exhibited negative correlations. These associations may reflect the concurrent changes in ginsenoside composition, phenolic and flavonoid contents, and antioxidant activity during HTHPS treatment. This observation is consistent with previous studies reporting associations between secondary metabolite contents and antioxidant activity [43,44,45].

The BI was positively associated with several rare ginsenosides and antioxidant activity indicators, while showing negative associations with major ginsenosides. These patterns suggest that BI may be related to the overall extent of compositional changes during HTHPS processing. Nevertheless, further validation using independent samples and additional analyses is required before BI can be established as a reliable indicator of processing intensity or product quality.

## 3. Materials and Methods

### 3.1. Materials

In October 2025, fresh four-year-old American ginseng roots were procured from a cultivation base in Wendeng District, Weihai City, Shandong Province, China. They were identified as American ginseng (*Panax quinquefolium* L.) of the Araliaceae family in accordance with the standards for morphological identification in the *Chinese Pharmacopoeia* (2025 Edition). For experimental purposes, samples were rigorously screened to ensure an average root weight of 58 ± 5 g (fresh weight), a moisture content of approximately 60~65%, a processing yield of approximately 39% (Table S1), and the absence of pests, diseases, or mechanical damage.

### 3.2. Processing

For each of the three independent sample-preparation batches, three fresh American ginseng roots were selected, washed, and trimmed. Each root was then longitudinally divided into five equal segments, with one segment from each root randomly assigned to each of the five treatment groups. One group served as the control (freeze-dried; Con), while the remaining four groups received 1–4 HTHPS cycles. The samples were wrapped in gauze and aluminum foil and subjected to high-temperature, high-pressure steaming at 120 °C, at which point the gauge pressure reached 0.105 MPa for 90 min per cycle using equipment (SQL1010C, Shenzhen Hongrui Automation Equipment Co., Ltd., Shenzhen, China). The treatment conditions were based on previously reported procedures [46]. Each treatment cycle comprised heating, holding, and cooling stages.

### 3.3. Color Analysis

The color of American ginseng was measured using a colorimeter (CR-400, Konica Minolta, Osaka, Japan). The color difference (ΔE) and browning index (BI) were calculated as described previously [47,48].

### 3.4. Sample Extraction

The samples were pulverized into a fine powder and subsequently passed through a 100-mesh sieve. A 200 mg aliquot of the powdered sample was extracted once with 20 mL of 80% methanol. This mixture underwent vortexing and sonication at temperatures maintained below 40 °C for a duration of 1 h. Following this, the mixture was allowed to cool to room temperature and was centrifuged at 7000× *g* for 10 min. The collected supernatant was filtered through a 13 mm Nylon 66 filter membrane (pore size 0.22 μm), and the filtrate was used for HPLC and subsequent analysis [28].

### 3.5. Determination of Ginsenoside Content

The quantification of ginsenoside content was conducted utilizing high-performance liquid chromatography (HPLC) as described by [49]. The analytical procedure employed an LC-20AT system, which was integrated with a SIL-20A autosampler, an SPD-20A UV detector, and a CTO-20A column oven (Shimadzu Corporation, Tokyo, Japan). The mobile phase consisted of water (A) and acetonitrile (B), with the following gradient: 17–23% B (0–30 min), 23–24% B (30–35 min), 24–32% B (35–45 min), 32–44% B (45–48 min), 44% B (48–52 min), 44–55% B (52–65 min), 55–100% B (65–85 min), 100% B (85–95 min), and 100–17% B (95–105 min). A Kinetex C18 column (100 mm × 4.6 mm, 2.6 µm; Torrance, CA, USA) was used and the temperature was maintained at 35 °C. The flow rate was 1.0 mL/min and the injection volume was 10 μL. The following ginsenosides were analyzed: Rg1, Re, Rh1, Rg2, Rb1, Rc, Rb2, Rb3, Rd, Rg6, F4, Rk3, Rh4, Rg3, Rk1, Rg5, and Rh2. The compounds were detected at 203 nm, identified by comparison of their retention times with those of the corresponding standards (Chengdu Alfa Biotechnology Co., Ltd., Chengdu, China), and quantified using the corresponding calibration curves (Tables S2 and S3).

### 3.6. Determination of TPC and TFC

TPC and TFC were determined according to Park et al. [50] and Liu et al. [51], with slight modifications. For TPC, 0.5 mL of the standard or sample solution was mixed with 0.5 mL of 80% methanol, 2.5 mL of distilled water, and 0.25 mL of 50% Folin–Ciocalteu reagent. After 5 min, 0.5 mL of 5% sodium carbonate was added. The mixture was incubated in the dark for 1 h. Absorbance was measured at 725 nm. TPC was expressed as mg gallic acid equivalents (GAE)/g dry weight (d.w.).

For TFC, 0.5 mL of the standard or sample solution was mixed with 2.8 mL of distilled water, 2.0 mL of 80% methanol, 0.1 mL of 10% aluminum chloride, and 0.1 mL of 1.0 mol/L potassium acetate. The mixture was vortexed and incubated in the dark for 40 min. Absorbance was measured at 415 nm. TFC was expressed as mg quercetin equivalents (QE)/g dry weight (d.w.).

### 3.7. Determination of Antioxidant Activity

#### 3.7.1. DPPH Radical Scavenging Activity

DPPH radical-scavenging activity was determined according to Oh et al. [52], with minor modifications. Weigh 1 g of each sample treated with different HTHPS treatments, add 25 mL of 80% methanol solution to a 50 mL centrifuge tube, and sonicate at 40 °C for 1 h. Subsequently, centrifuge the samples at 6500 r/min for 15 min. Collect the supernatant and filter it through a 0.22 μm filter membrane. Mix 1 mL of the sample solution at different concentrations with 3 mL of 0.1 mM DPPH solution (prepared with 80% methanol); after reacting for 30 min at room temperature in the dark, measure the absorbance at a wavelength of 517 nm. The half-inhibitory concentration (IC_50_) refers to the concentration at which the sample scavenges 50% of DPPH.

#### 3.7.2. ABTS^+^ Radical Scavenging Activity

ABTS^+^ radical-scavenging activity was determined according to Lim et al. [53], with minor modifications. Preparation of the ABTS^+^ Working Solution: Mix equal volumes of a 7 mM ABTS solution and a 2.45 mM potassium persulfate solution. Allow the mixture to react in the dark at room temperature for 12–16 h to obtain the ABTS^+^ stock solution. Before use, dilute with 80% methanol until the absorbance at 734 nm is 0.70 ± 0.02; this is the ABTS^+^ working solution. Prepare the sample solutions by following the procedure described in Section 3.7.1 above. Place 0.5 mL of sample solutions of different concentrations into test tubes and add 5 mL of the prepared ABTS solution to each. After allowing the reaction to proceed for 10 min at room temperature in the dark, measure the absorbance at a wavelength of 734 nm. The half-inhibitory concentration (IC_50_) represents the sample concentration required to scavenge 50% of the ABTS^+^ free radicals.

#### 3.7.3. ·OH Radical Scavenging Activity

·OH radical-scavenging activity was determined according to Jiang et al. [54], with minor modifications. Prepare the sample solutions by following the procedure described in Section 3.7.1 above. Take 0.1 mL of sample solutions of different concentrations and add, in sequence, 0.1 mL of 6.0 mmol/L ferrous sulfate solution and 0.1 mL of 6.0 mmol/L hydrogen peroxide solution. Vortex the mixture to homogenize it, incubate for 10 min, and then add 0.1 mL of 6.0 mmol/L salicylic acid solution. Incubate the mixture in the dark for 30 min, then measure the absorbance at a wavelength of 510 nm. The half-inhibitory concentration (IC_50_) represents the sample concentration required to scavenge 50% of the ·OH free radicals.

### 3.8. Data Analysis

The data were analyzed utilizing SPSS version 19.0. A one-way analysis of variance (ANOVA), followed by Tukey’s HSD test, was employed to identify significant differences. Pearson correlation analysis was conducted to evaluate the relationships between bioactive components and antioxidant indices. Statistical significance was defined at a threshold of *p* < 0.05. Each treatment included three biological replicates, and all measurements were performed in triplicate. The results are expressed as mean ± standard error. Graphical representations were generated using Origin 2024.

## 4. Conclusions

The application of HTHPS facilitated the transformation of major ginsenosides into rare ginsenosides and augmented the extractable polyphenol and flavonoid concentrations in American ginseng. Specifically, HTHPS-3 promoted the accumulation of several key rare ginsenosides, such as Rg3, Rk1, Rg5, Rh1, F4, and Rg6. In contrast, HTHPS-2 exhibited superior DPPH and ABTS^+^ radical-scavenging activities, whereas HTHPS-3 demonstrated the highest ·OH radical-scavenging activity. Correlation analysis revealed significant associations between polyphenols, flavonoids, and rare ginsenosides with antioxidant activity. Subsequent treatment with HTHPS-4 resulted in a reduction in several bioactive components and a decrease in antioxidant capacity. The optimal number of HTHPS cycles depends on the specific processing objectives rather than a single overall criterion. HTHPS-2 was more suitable when maximizing TPC and DPPH/ABTS^+^ radical-scavenging activities was the primary objective. In contrast, HTHPS-3 offered greater advantages for rare ginsenoside accumulation, particularly Rg3, Rk1, and Rg5, and for ·OH radical-scavenging capacity. These differences indicate that the responses of individual bioactive components and antioxidant indicators to repeated HTHPS treatment are not identical. Therefore, selecting the number of HTHPS treatment cycles should be guided by the intended application and the specific quality attributes to be optimized.

## Figures and Tables

**Table 1:** Scavenging capacity (IC_50_) of samples treated with different HPHTS methods against DPPH, ABTS^+^, and ·OH radicals.

Treatment Group	IC_50_ (mg/mL)		
	DPPH Radical-Scavenging Ability	ABTS^+^ Radical-Scavenging Ability	·OH Radical-Scavenging Ability
Con	22.35 ± 0.47 a	4.22 ± 0.16 a	30.08 ± 0.49 a
HTHPS-1	6.22 ± 0.13 b	2.06 ± 0.01 b	11.52 ± 0.08 b
HTHPS-2	3.50 ± 0.02 e	1.46 ± 0.05 d	10.27 ± 0.25 c
HTHPS-3	3.89 ± 0.05 d	1.49 ± 0.02 d	9.56 ± 0.19 d
HTHPS-4	4.34 ± 0.11 c	1.63 ± 0.01 c	10.05 ± 0.27 cd

Letters a–e above the line charts indicate significant differences at *p* < 0.05 using Tukey’s HSD test.

## Data Availability

Data is contained within the article and Supplementary Materials.

## Supplementary Materials

The following supporting information can be downloaded at: https://www.mdpi.com/article/10.3390/molecules31203706/s1, Table S1: Moisture content and dry weight ratio of American ginseng under different HTHPS treatments. Table S2: Linear Regression Fitting Equations for 17 Saponins in American Ginseng. Table S3: Recovery rates of 17 ginsenosides.
