# Supplementary material for: The Impact of High-Temperature and High-Pressure Steaming Treatment on Bioactive Compounds and Antioxidant Activity in American Ginseng
Source: Molecules. 2026 Oct 16;31(20):3706. doi: 10.3390/molecules31203706 (PMC13707937; doi:10.3390/molecules31203706)
Supplement: Supplementary file 1 [file molecules-31-03706-s001.zip › molecules-4600557-supplementary.pdf]

Supplementary Material

Table S1. Moisture content and dry weight ratio of American ginseng under different HTHPS treatments

Table S2. Linear Regression Fitting Equations for 17 Saponins in American Ginseng

Table S3. Recovery rates of 17 ginsenosides

Table S1. Moisture content and dry weight ratio of American ginseng under different HTHPS treatments

Treatment Group	Fresh Weight (g)	Dry Weight (g)	Processing yield (%)	RSD (%)
Con	12.62 ± 1.89	4.76 ± 0.66	37.81 ± 0.92	1.48
HTHPS-1	10.04 ± 0.33	3.96 ± 0.03	39.43 ± 1.50	2.48
HTHPS-2	11.46 ± 0.60	4.50 ± 0.41	39.23 ± 1.13	1.86
HTHPS-3	12.25 ± 1.50	4.75 ± 0.54	38.82 ± 0.81	1.33
HTHPS-4	12.23 ± 0.39	4.80 ± 0.27	39.23 ± 1.51	2.48

Results are expressed as mean ± standard deviation. A two-factor analysis of variance or a linear mixed model was used to analyze treatment effects, with treatment as a fixed effect and the main root as a random effect (block). A $P < 0.05$ was considered statistically significant.

Table S2. Linear Regression Fitting Equations for 17 Saponins in American Ginseng

Ingredients	Regression Equation	R^2	LOD (mg/L)	LOQ (mg/L)
Rg1	Y=4062.3x+3704.1	0.9999	1.20	3.62
Re	Y=3810.7x+861.12	1.0000	0.44	1.32
Rh1	Y=5992.3x+3399.7	1.0000	0.58	1.75
Rg2	Y=4702.8x+2638.2	1.0000	0.56	1.71
Rb1	Y=2870.8x+1708.9	1.0000	0.52	1.57
Rc	Y=3139.4x+1464.5	1.0000	0.43	1.31
Rb2	Y=3050.7x+1441.8	0.9999	0.29	0.89
Rb3	Y=3126.3x+503.21	0.9999	0.91	2.76
Rd	Y=3321.1x+848.54	0.9976	5.98	18.13
Rg6	Y=9587.8x-50.667	0.9999	1.27	3.86
F4	Y=12813x+1522.7	1.0000	0.37	1.12
Rk3	Y=13555x+2685	1.0000	0.37	1.11
Rh4	Y=12791x+2326.1	0.9999	0.99	2.99
Rg3-s	Y=4526.6x+3482.6	0.9999	0.95	2.87
Rk1	Y=9439.8x+8278.4	0.9999	0.97	2.93
Rg5	Y=3665.8x+1616.9	1.0000	0.73	2.20
Rh2	Y=6376.1x+4979.7	0.9998	1.62	4.90

Table S3. Recovery rates of 17 ginsenosides

Ingredients	Spiked Recovery(%)	RSD(%)
Rg1	99.02	0.12
Re	101.94	1.01
Rh1	103.30	0.67
Rg2	103.89	1.86
Rb1	98.19	0.93
Rc	101.00	0.42
Rb2	103.91	1.16
Rb3	103.39	0.85
Rd	100.54	0.73
Rg6	99.80	0.30
F4	100.50	1.25
Rk3	101.53	0.66
Rh4	99.66	0.70
Rg3-s	99.19	0.60
Rk1	100.32	1.05
Rg5	103.77	0.71
Rh2	99.78	0.62
